# Cardiovascular Safety and Possible Benefit of a 5-Alpha Reductase Inhibitor among Benign Prostatic Hyperplasia Patients, A Nationally Representative Cohort of Korean Men

**DOI:** 10.3390/jcm8050733

**Published:** 2019-05-22

**Authors:** Jooyoung Chang, Seulggie Choi, Kyuwoong Kim, Sang Min Park

**Affiliations:** 1 Department of Biomedical Sciences, Seoul National University Graduate School, Biomedical Science Building 117, 103 Daehak-ro, Jongro-gu, Seoul 03080, Korea; joomyjoo@gmail.com (J.C.); seulggie@gmail.com (S.C.); kwkim238@gmail.com (K.K.); 2 Department of Family Medicine, Seoul National University Hospital, 103 Daehak-ro, Jongro-gu, Seoul 03080, Korea

**Keywords:** benign prostatic hyperplasia, 5-alpha reductase inhibitor, drug safety, retrospective cohort, cardiovascular disease, myocardial infarction, stroke

## Abstract

Several studies suggest that 5-alpha reductase inhibitors (5ARIs) may be associated with elevated risk of cardiovascular disease (CVD). We investigated the association of 5ARI exposure and CVD incidence using the National Health Insurance Service-Health Screening Cohort, a nationally representative population-based sample of Koreans. We calculated the 4-year cumulative exposure to 5ARI for 215,003 men without prior 5ARI use. Participants were followed from January 1st, 2008 to December 31st, 2015. A subcohort of newly diagnosed benign prostatic hyperplasia (BPH) patients during 2004–2010 was also analyzed. The primary study outcome was CVD and secondary outcomes were myocardial infarction (MI) or stroke. Hazard ratios (HRs) were estimated using Cox proportional hazards models adjusted for conventional risk factors. In both the main cohort and BPH subcohort, the use of any 5ARI did not increase the risk of cardiovascular disease (HR = 1.06; 95% CI = 0.91–1.23; HR = 0.95; 95% CI = 0.88–1.03; respectively). Furthermore, as an unexpected finding, a dose-analysis among the BPH subcohort showed that the highest tertile of 5ARI exposure reduced the risk of CVD (HR = 0.82; 95% CI = 0.72–0.92; *p*-trend = 0.001), MI (HR = 0.69; 95% CI = 0.50–0.95), and stroke (HR = 0.84; 95% CI = 0.72–0.98) compared to non-users. Among men and BPH patients, 5ARI did not increase the risk of CVD. Among BPH patients, 5ARI use may reduce the risk CVD.

## 1. Introduction

The long-term cardiovascular safety of 5-alpha reductase inhibitor (5ARI) has not been adequately addressed in the current literature. 5ARI inhibits prostatic growth by preventing the conversion of testosterone (T) to its more potent form dihydrotestosterone (DHT) [1]. Finasteride and dutasteride, currently available forms of 5ARI, suppresse DHT levels by approximately 75% and 90%, respectively [2,3]. For symptomatic benign prostatic hyperplasia (BPH), 5ARI is commonly prescribed with or without alpha-adrenergic blockers (alpha-blockers) [4].

Despite the widespread use of 5ARI, studies have conflicting results over the cardiovascular safety of 5ARI. Some studies highlight benefits of DHT in improving cardiovascular risk factors [5], suggesting 5ARI’s antiandrogenic effect may elevate cardiovascular disease (CVD) risk. Likewise, androgen deprivation therapy (ADT) in prostate cancer patients increased the risk of CVD by causing metabolic complications [6,7,8]. However, a recent study showed that both lower DHT and high DHT patients had a greater risk of incident CVD than normal DHT patients [9,10]. A randomized clinical trial (RCT) showed that dutasteride increased the risk of heart failure compared to placebo controls [11]. However, this finding was contested by a systematic review of 12 RCTs which showed no significant association [12]. A retrospective cohort analysis among a Taiwanese population found no significant risk of CVD associated with 5ARI users relative to non-users among BPH patients [13], but this study did not adjust for key cardiovascular risk factors such as tobacco use.

The cardiovascular risk of 5ARI use has not been addressed completely and, given its widespread use and lack of evidence for long-term effects, this merits further investigation. We aimed to determine the CVD risk of 5ARI using a population-based study using the Korean National Health Insurance Service database.

## 2. Materials and Methods

### 2.1. Study Sample

The Korean National Health Insurance Service-Health Screening Cohort (NHIS-HEALS) was used in this study [14,15]. NHIS-HEALS is a nationally representative cohort consisting of healthcare beneficiaries aged 40 through 79 years, sampled by a simple random sampling method of all health screening participants during years 2002 and 2003 in Korea. Insurance services are universal in Korea, and beneficiaries of 40 years or more are eligible for a free biennial health screening [14,15]. NHIS-HEALS contains information on demographics, insurance claims, health screening results, and death registries of participants from 2002 to 2015. The study was approved by the Institutional Review Board of Seoul National University Hospital (IRB number: E-1804-015-933). The ethics committee waived the need for participant consent, because all data were anonymized by the NHIS.

### 2.2. Study Design of the Main Cohort

**Figure 1:**
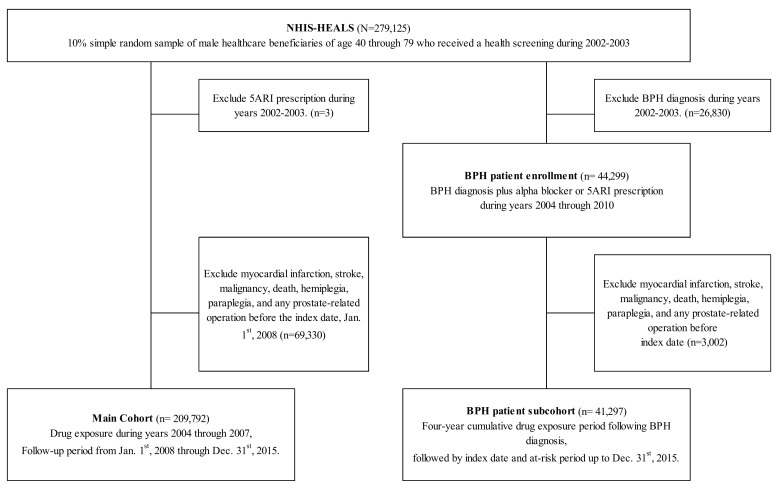
Study Design of the Male Cohort and Benign Prostatic Hyperplasia Subcohort.

For the analysis of cardiovascular risks of 5ARI among the general male population, we constructed a cohort of men enrolled on January 1st, 2004, after excluding those with 5ARI prescriptions in years 2002 and 2003 (Figure 1). The drug-exposure period was set to four years, 2004 through 2007. The index date was set to January 1st, 2008, before which time covariates were assessed. Those who died before the index date or had previous insurance claims for CVD, MI, stroke, or prostatic surgery were excluded. The follow-up period did not include the drug exposure period to avoid immortal time bias. Subsequently, patients were observed for event outcomes during the follow-up period from January 1st, 2008 through December 31st, 2015.

A detailed outline for the study design is depicted in Supplementary Figure S1.

### 2.3. Study Design of the BPH Patient Subcohort

For the analysis of 5ARI use among BPH patients, we constructed a fixed-entry subcohort of newly diagnosed BPH patients from years 2004 through 2010, after exclusion of BPH diagnosis in years 2002 and 2003 (Figure 1). To reduce the heterogeneity of BPH severity, we enrolled only patients who received a prescription of either alpha-blockers, 5ARIs, or both, under the diagnosis of BPH, using the International Classification of Diseases 10th revision (ICD-10) code, “N40.” Exposure to drugs was calculated during a 4-year period following enrollment, after which the index date marked the start of follow-up period. To avoid immortal time bias, the follow-up person-years did not include the four-year drug exposure period—none of the cumulated person-years were subject to immortal time. We assessed covariates for the 6-year period before the index date. Those who died or had insurance claims for CVD, MI, stroke, or prostatic surgery before the index date were excluded. Subsequently, during the follow-up period from the index date through December 31st, 2015, patients were observed for event outcomes.

### 2.4. Ascertainment of Event Outcomes

Starting from the index date, cohort enrollees were followed until the primary outcome of CVD and secondary outcomes of MI and stroke. We defined outcomes as two or more days of admission, or death due to CVD (Supplementary Table S1). The follow-up duration ended on the first incidence of a primary or secondary outcome or by death.

### 2.5. Drug Exposure

Drug exposures were calculated using the World Health Organization Anatomic Therapeutic Chemical Classification System (WHO-ATC) and its Daily Defined Dose (DDD) [16]. DDD is a standardized drug quantity measurement. For 5ARI, one DDD unit is defined as either 5 mg of finasteride or 0.5 mg of dutasteride [17].

The cumulative DDD (cDDD) of patients was determined during the drug-exposure period using patient prescriptions verified by pharmacy dispense. Those without any dispensing of 5ARI were considered non-users, those with dispensing of 5ARI were considered users. For the dose-response analysis, 5ARI users were divided into tertiles according to their cumulative doses. For the main cohort, we adjusted for the calendar year of the first prescription of 5ARI to adjust for changes in drug prescription patterns over time. For the BPH patient subcohort, we adjusted for the year of BPH diagnosis to adjust for changes in drug prescription patterns for treatment of BPH over time.

The prescription and dispensing of alpha-blocker, aspirin, non-aspirin non-steroidal anti-inflammatory drugs (NSAID), and HMG-CoA reductase inhibitors were also determined in the drug-exposure period. For purposes of adjustment, the cDDD of covariate drugs was divided into groups of non-users, 0 to 30, 30 to 180, and 180 or more cDDD. Because alpha-blockers are used for similar indications as 5ARI, cDDD of alpha-blockers was divided into non-users and tertiles of users.

### 2.6. Determination of Covariates

Comorbidities were determined by using the insurance claims data and by laboratory measurement from the most recent health screening before the index date. Those with fasting blood glucose level of 126 mg/dL or more, or previous diagnosis of diabetes with prescriptions of glucose lowering drugs were considered diabetic. Total cholesterol of 240 mg/dL was considered hypercholesteremic. Those with systolic blood pressure of 140 mmHg or more, or diastolic blood pressure of 90 mmHg or more were considered hypertensive. Other comorbidities such as atrial fibrillation, acute urinary retention, angina, and Charlson Comorbidity Index (CCI) were assessed using the ICD-10 codes of the insurance claims database as outlined in Supplementary Table S1. The number of outpatient visits was determined by the number of outpatient insurance claims.

For the main cohort, the diagnosis of any ICD-10 code, “N40,” before the index date was considered a diagnosis of BPH. For the analysis of the main cohort, BPH diagnosis status was used as a covariate. For the BPH patient subcohort, only those with a diagnosis of ICD-10 code, “N40,” plus prescription of alpha-blockers or 5ARI were considered BPH patients. For the subcohort, BPH diagnosis was used as the enrollment criteria rather than a covariate.

Body mass index, smoking habit, alcohol consumption frequency, and exercise frequency were assessed for each patient using the latest health screening. Age and sex were determined by insurance eligibility, and socioeconomic status (SES) was determined by quartiles of the insurance premium.

### 2.7. Statistical Analysis

We conducted a chi-square test between 5ARI use and covariates. Using a multivariate Cox proportional hazards model adjusted for age, hypertension, diabetes, high cholesterol, body mass index, smoking habit, alcohol consumption frequency, exercise frequency, socioeconomic status in quartiles, acute urinary retention, atrial fibrillation or flutter, angina, Charlson Comorbidity Index, outpatient visits, alpha-blocker use, aspirin use, NSAID use, HMG-CoA reductase inhibitor use, benign prostatic hyperplasia, and year of first 5ARI prescription or year of BPH diagnosis, we estimated hazard ratios of 5ARI users relative to non-users. For a dose-response analysis of 5ARI users, we performed a multivariate Cox proportional hazards analysis of the tertiles of users with reference to non-users. The analysis of the p-for-trend was determined by treating the categorical variable of non-users, first tertile of users, second tertile of users, and third tertile of users as numerical values of 0, 1, 2, and 3, respectively. To assess the risks among low-risk populations, we performed a stratified analysis by major risk factors such as age, hypertension, diabetes, and concurrent aspirin use. All data mining and statistical analysis were implemented using SAS®, version 9.4 (SAS Institute, Cary, NC, USA).

## 3. Results

Among 279,125 male beneficiaries who received a health screening during 2002–2003, a total of 209,792 males were observed from the index date after exclusion of prior drug use or MI, stroke, malignancy, hemiplegia, paraplegia, death, or prostate operations (Figure 1). For the BPH patient subcohort, 48,140 patients were enrolled between 2004 through 2010, and observed for outcomes following four years of drug exposure (Figure 1).

The characteristics of the main cohort and the BPH patient subcohort showed that 5ARI use was associated with older age, never smoking, less frequent alcohol consumption, lower SES, acute urinary retention, higher CCI, aspirin use, NSAID use, HMG-CoA reductase inhibitor use, alpha-blocker use, and more frequent outpatient visits (all *p* values < 0.05; Table 1).

The use of any 5ARI did not significantly increase the risk of CVD in both the main cohort (HR = 1.06; 95% CI = 0.91–1.23) and the BPH patient subcohort (HR = 0.95; 95% CI = 0.88–1.03). 5ARI use was not significantly associated with MI or stroke (Table 2). These outcomes did not change when stratified by major cardiovascular risk factors (Supplementary Table S2).

**Figure 2:**
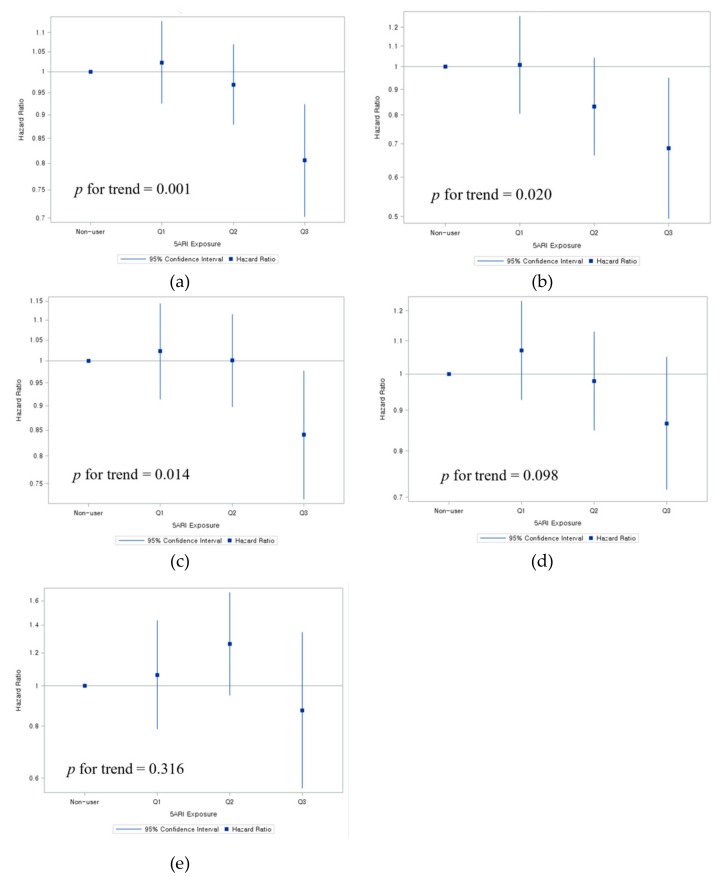
Hazard ratios of tertiles of 5ARI users among the BPH patient subcohort. (**a**) Hazard Ratio for Cardiovascular Disease. (**b**) Hazard Ratio for Myocardial Infarction. (**c**) Hazard Ratio for Stroke. (**d**) Hazard Ratio for Ischemic Stroke. (**e**) Hazard Ratio for Hemorrhagic Stroke. Hazard ratios were estimated using a multivariate cox proportional hazard model (Wald χ2 test *p* value<0.001) adjusted for age, hypertension, diabetes, high cholesterol, body mass index, smoking habit, alcohol consumption frequency, exercise frequency, socioeconomic status in quartiles, benign prostatic hyperplasia, acute urinary retention, atrial fibrillation or flutter, angina, Charlson Comorbidity Index, outpatient visits, alpha-blocker use, aspirin use, NSAID use, HMG-CoA reductase inhibitor use, and year of BPH diagnosis.

An analysis of the dose-response showed a null association in the main cohort (Supplementary Table S3). However, in the BPH patient subcohort, the highest tertile of 5ARI users experienced a significant reduction of CVD (HR = 0.81; 95% CI = 0.70–0.92), MI (HR = 0.69; 95% CI = 0.50–0.95), and stroke (HR = 0.84; 95% CI = 0.72–0.98) (Figure 2, Supplementary Table S3). When stratified by aspirin use and age, mostly aspirin non-users and older patients experienced a reduced risk of CVD and stroke (Table 3).

## 4. Discussion

In this population-based study, the use of 5ARI did not increase the risk of CVD, MI, and stroke among the general male population among BPH patients. As an unexpected finding a dose-response analysis of the BPH patient subcohort suggested a significant trend in reduction of CVD risk with greater use of 5ARI, with the highest tertile of 5ARI users experiencing significant risk reduction referent to non-users.

A previous population-based cohort study showed that androgen deprivation therapy (ADT), which reduces serum T, DHT, and other androgens [18,19], increased the risk of MI and stroke among prostate cancer patients [8], possibly due to harmful metabolic effects [6,7]. With considerable evidence supporting the protective cardiovascular properties of DHT, concerns were raised as to whether a reduction in DHT was part of the hazard. DHT is associated with better cardiovascular risk factors [20], lower ischemic heart disease mortality [5], and endothelial health via an anti-inflammatory effect and an inhibitory effect on foam cell formation [21].

Despite these concerns, we found no significant increase in cardiovascular risk among 5ARI users compared to non-users, perhaps due to some key differences between ADT and 5ARI treatment. First, ADT includes treatments such as gonadotropin-releasing hormone (GnRH) agonists, GnRH antagonists, and orchiectomy [22], which suppresse a broad spectrum of androgens including testosterone (T), DHT, dehydroepiandrosterone (DHEA), adrenocorticotropic hormone ACTH, and androstenedione [18]. Evidence suggests CVD risk may be due to mechanisms involving activation of GnRH receptors found in T lymphocytes [22] and increased levels of FSH [23] by GnRH agonists, while clear mechanisms linking lowered DHT levels and CVD risk are unclear. Second, the magnitude of DHT suppression by ADT is greater than that by 5ARI treatment [2,3,18,24], which suggests the excessive suppression of DHT by ADT may have caused the increased risk.

Among BPH patients, users of 5ARI within 4 years of diagnosis benefited from a reduced risk of cardiovascular disease. Since BPH patients generally have higher supraphysiologic levels of DHT [25,26,27] and previous studies show that high DHT patients were at a higher risk of CVD than normal DHT patients [9,10], a correction of DHT levels by 5ARI use may have caused the benefit. This may explain why 5ARI did not have any protective effect in the main cohort, where the reference group includes non-BPH participants who are more likely to have physiologic levels of DHT. Moreover, the protective effects of 5ARI were present among only the highest tertile of users, and only present among older men and aspirin non-users, which suggests a possible link between 5ARI use and thrombogenicity [28,29]. However, the protective effects of 5ARI in this study are an incidental finding and actual DHT levels were not determined, which warrants further investigation.

### 4.1. Strengths

Our study has several strengths. First, our cohort is large, with 209,792 men for the main cohort and 48,140 men for the BPH patient subcohort. Second, our cohort comprises of complete reports of any insured hospital and pharmacy visits, and represents the general population of insured, health screened Koreans. On the contrary, reporting of cardiovascular adverse events was unclear in previous RCTs [30]. Third, the follow-up duration of our data enables us to test potential risks of long-term drug use. In contrast, RCTs inherently have limited study durations and size [12] and enroll patients that may differ from real-world patients [31].

### 4.2. Limitations

Our data were collected for administration of insurance claims and reimbursement. Thus, we do not have access to information regarding medication adherence or uninsured indications (i.e., alopecia) for 5ARI prescriptions which may have played a part in cardiovascular outcomes. With the data available, however, we made sure clinical prescriptions were verified by with pharmacy dispensing, which reflects drug possession, not just indications for use. Furthermore, patterns of diagnosis and prescription may be affected by changes in insurance policies over time. However, we made sure to adjust for the calendar year for diagnoses and incorporated measurements of fasting blood glucose, blood pressure, and total cholesterol for key covariates such as diabetes, hypertension, and high cholesterol. The severity of BPH is unknown, which may affect prescription patterns. However, we tried to minimize the heterogenicity of BPH patients by enrolling only those patients with prescriptions of alpha-blocker or 5ARI.

## 5. Conclusions

The use of 5ARI did not significantly increase the risk of CVD, MI, or stroke with reference to non-users in both the main cohort and the BPH patient subcohort. 5ARI was dose-responsively protective for CVD, MI, or stroke among BPH patients, possibly due to normalization of DHT levels. Therefore, clinicians should not actively refrain from 5ARI prescription for BPH. Further studies of 5ARI use, with measurements of serum DHT, are warranted.

## Figures and Tables

**Table 1:** Characteristics of the Main Cohort and BPH patient subcohort by 5ARI use.

%, Unless Otherwise Stated	Main Cohort			BPH Patient Subcohort		
	Non-User	User	*p* Value *	Non-User	User	*p* Value *
*n* (number)	200,641	9151		20,749	20,548	
Age			<0.001			<0.001
40–50	33.4	9.3		15.4	8.4	
50–60	40.4	31.1		39.8	32.3	
60–70	19.2	37.6		29.2	36.6	
≥70	7.0	22.1		15.6	22.7	
Body Mass Index, kg/m2			0.388			0.130
<23	35.9	35.2		35.8	35.0	
23–25	28.8	29.0		28.9	29.7	
≥25	35.3	35.7		35.3	35.3	
Smoking Habit			<0.001			<0.001
Never	39.2	49.2		40.7	44.5	
Past	14.7	16.6		32.3	31.5	
Current	41.9	29.9		24.4	21.5	
Alcohol Consumption, per week			<0.001			<0.001
Fewer than once	52.2	59.0		46.6	51.3	
1–2	27.3	22.4		31.6	28.7	
≥3	19.2	17.1		21.1	19.2	
Exercise Frequency, per week			<0.001			0.101
Fewer than once	46.8	46.9		12.3	12.4	
1–2	30.5	26.3		35.9	36.8	
≥3	20.0	24.4		51.8	50.8	
Socioeconomic Status, quartiles			<0.001			<0.001
Q1, Lowest	23.7	26.3		24.7	27.2	
Charlson Comorbidity Index			<0.001			<0.001
≥3	9.8	20.3		45.2	49.8	
Outpatient Visits, tertiles			<0.001			<0.001
Q3, most frequent	33.2	67.9		30.2	39.2	
High cholesterol	12.4	13.4	0.001	9.4	8.8	0.064
Hypertension	35.8	38.4	<0.001	22.6	23.5	0.027
Benign Prostatic Hyperplasia	10.6	96.5	<0.001	100	100	
Diabetes	11.5	14.2	<0.001	13.0	13.0	0.902
Atrial Fibrillation or Flutter	1.2	2.5	<0.001	1.0	1.0	0.869
Angina	9.2	18.0	<0.001	6.2	6.3	0.689
Acute Urinary Retention	0.1	1.6	<0.001	0.4	0.7	<0.001
Alpha-blocker use †	2.3	48.2	<0.001	45.5	65.1	<0.001
Aspirin use †	9.9	18.5	<0.001	25.8	29.5	<0.001
Non-aspirin NSAID use †	24.2	45.0	<0.001	48.5	54.7	<0.001
HMG-CoA reductase inhibitor use †	7.0	11.7	<0.001	22.0	23.9	<0.001

Abbreviations: 5ARI, 5-alpha reductase inhibitor; NSAID, non-steroidal anti-inflammatory drug. * of χ2 test with 5ARI use (user vs. non-user). † ≥30 cDDD.

**Table 2:** Hazard ratios of 5ARI users vs. non-users.

	Exposure to 5ARI	
	Non-User (0 cDDD)	User (≥1 cDDD)
**Main cohort**		
CVD (Stroke or MI)		
Cases	10,101	744
aHR * (95% CI)	1(ref.)	1.06 (0.91–1.23)
Myocardial Infarction		
Cases	2387	148
aHR * (95% CI)	1(ref.)	1.11 (0.81–1.53)
Stroke		
Cases	7682	587
aHR * (95% CI)	1(ref.)	1.04 (0.88–1.23)
**BPH patient subcohort**		
CVD (Stroke or MI)		
Cases	1348	1536
aHR * (95% CI)	1(ref.)	0.95 (0.88–1.03)
Myocardial Infarction		
Cases	285	282
aHR * (95% CI)	1(ref.)	0.86 (0.72–1.02)
Stroke		
Cases	1067	1256
aHR * (95% CI)	1(ref.)	0.97 (0.89–1.06)

Abbreviations: 5ARI, 5-alpha reductase inhibitor; cDDD, cumulative daily defined dose; CVD, cardiovascular disease; aHR, adjusted hazard ratio; ref., referent; Q, quantile (tertile). * Using a multivariate cox proportional hazard (Wald χ^2^ test *p* value<0.001) adjusted for age, hypertension, diabetes, high cholesterol, body mass index, smoking habit, alcohol consumption frequency, exercise frequency, socioeconomic status in quartiles, acute urinary retention, atrial fibrillation or flutter, angina, Charlson Comorbidity Index, outpatient visits, alpha-blocker use, aspirin use, NSAID use, and HMG-CoA reductase inhibitor use. Main cohort was additionally adjusted for benign prostatic hyperplasia and year of first 5ARI prescription. BPH patient subcohort was additionally adjusted for year of BPH diagnosis.

**Table 3:** Hazard Ratios of 5ARI users (tertiles) vs. non-users of BPH patient subcohort, stratified by aspirin use and age.

BPH Patient Subcohort		Non-User	5ARI Exposure			
			Q1 of User (1–42 cDDD) aHR * (95% CI)	Q2 of User (43–216 cDDD) aHR * (95% CI)	Q3 of User (≥217 cDDD) aHR * (95% CI)	
Aspirin User (>30 cDDD)	CVD	1(ref.)	0.95 (0.81–1.10)	0.93 (0.80–1.09)	0.90 (0.77–1.06)	
	MI	1(ref.)	0.85 (0.62–1.17)	0.69 (0.50-0.96)	0.78 (0.56–1.09)	
	Stroke	1(ref.)	0.98 (0.82–1.16)	1.01 (0.85–1.20)	0.95 (0.79–1.13)	
Aspirin Non-user	CVD	1(ref.)	1.00 (0.87–1.16)	1.02 (0.87–1.19)	0.74 (0.61–0.89)	
	MI	1(ref.)	1.19 (0.84–1.68)	0.81 (0.53–1.22)	0.72 (0.45–1.16)	
	Stroke	1(ref.)	0.96 (0.82–1.13)	1.05 (0.89–1.25)	0.74 (0.61–0.91)	
Age ≥60 years	CVD	1 (ref.)	1.01 (0.90–1.13)	0.98 (0.87–1.11)	0.81 (0.71–0.92)	
	MI	1 (ref.)	0.97 (0.73–1.27)	0.85 (0.63–1.15)	0.69 (0.50–0.96)	
	Stroke	1 (ref.)	1.01 (0.89–1.15)	1.00 (0.88–1.14)	0.84 (0.73–0.97)	
Age <60 years	CVD	1 (ref.)	1.06 (0.86–1.32)	1.03 (0.82–1.28)	0.95 (0.73–1.26)	
	MI	1 (ref.)	1.13 (0.75–1.69)	0.66 (0.41–1.06)	1.08 (0.66–1.77)	
	Stroke	1 (ref.)	1.04 (0.81–1.35)	1.18 (0.92–1.52)	0.90 (0.65–1.26)	

Abbreviations: 5ARI, 5-alpha reductase inhibitor; CVD, cardiovascular disease; MI, myocardial infarction; aHR, adjusted hazard ratio; ref., referent. * Using a multivariate cox proportional hazard (Wald χ^2^ test *p* value<0.001) adjusted for age, hypertension, diabetes, high cholesterol, body mass index, smoking habit, alcohol consumption frequency, exercise frequency, socioeconomic status in quartiles, benign prostatic hyperplasia, acute urinary retention, atrial fibrillation or flutter, angina, Charlson Comorbidity Index, outpatient visits, alpha-blocker use, aspirin use, NSAID use, HMG-CoA reductase inhibitor use, and year of BPH diagnosis.

## Supplementary Materials

The following are available online at https://www.mdpi.com/2077-0383/8/5/733/s1, Supplementary Table S1: Summary of definitions of outcomes, exclusion criteria, and comorbidities., Supplementary Table S2: Hazard ratios of 5ARI users vs. non-users after stratification by cardiovascular risk factors, Supplementary Table S3: Hazard ratios of tertiles of 5ARI users vs. non-users, Supplementary Figure S1: Diagram of the male cohort and benign prostatic hyperplasia subcohort study designs.
