# Supplementary material for: Cardiovascular Safety and Possible Benefit of a 5-Alpha Reductase Inhibitor among Benign Prostatic Hyperplasia Patients, A Nationally Representative Cohort of Korean Men
Source: J Clin Med. 2019 May 22;8(5):733. doi: 10.3390/jcm8050733 (PMC6572349; doi:10.3390/jcm8050733)
Supplement: Supplementary file 1 [file jcm-08-00733-s001.pdf]

SUPPLEMENTAL MATERIAL.

Supplementary Table 1. Summary of definitions of outcomes, exclusion criteria, and comorbidities.

Variable Type	Name of variable		Definition (ICD-10 codes plus other requirements)
Event Outcomes	Primary Outcome	Cardiovascular Disease	Admission for I21-I24, I60-I69 for two or more days<br>OR death due to I21-I24, I60-I69
	Secondary Outcomes	Myocardial Infarction	Admission for I21-I24 for two or more days<br>OR Death due to I21-I24
		Total Stroke	Admission for I60-I69 for two or more days<br>OR Death due to I60-I69
		Ischemic Stroke	Admission for I63 for two or more days<br>OR Death due to I63
		Hemorrhagic Stroke	Admission for I60-I62 for two or more days<br>OR Death due to I60-I62
Exclusion Criteria	Cardiovascular Disease		I21-I24, I60-I69
	Malignancy		C00-C97
	Hemiplegia, Paraplegia		G81, G820, G821, G822
	Prostate Operation		Insurance claims procedure codes
Comorbidities	Charlson Comorbidity Index		ICD-10 codes for congestive heart failure, peripheral vascular disease, dementia, pulmonary disease, connective tissue disorder, peptic ulcer, liver disease, renal disease, severe liver disease, and HIV.
	Hypertension		Blood pressure greater than SBP 140 mmHg or DBP 90 mmHg measured at the latest health screening before the index date.
	Diabetes		E10, E11, E12, E13, E14 plus any prescription of drugs used for treatment of diabetes, or 126 mg/dL or higher fasting blood glucose measured at the latest health screening before the index date.
	High cholesterol		240 mg/dL or higher total cholesterol measured at the latest health screening before the index date.
	Acute urinary retention		R33
	BPH		N40
	Atrial Fibrillation/Flutter		I48
	Angina or chronic ischemic heart disease		I20, I251, I258, I259

Supplementary Figure 1. Diagram of the male cohort and benign prostatic hyperplasia subcohort study designs

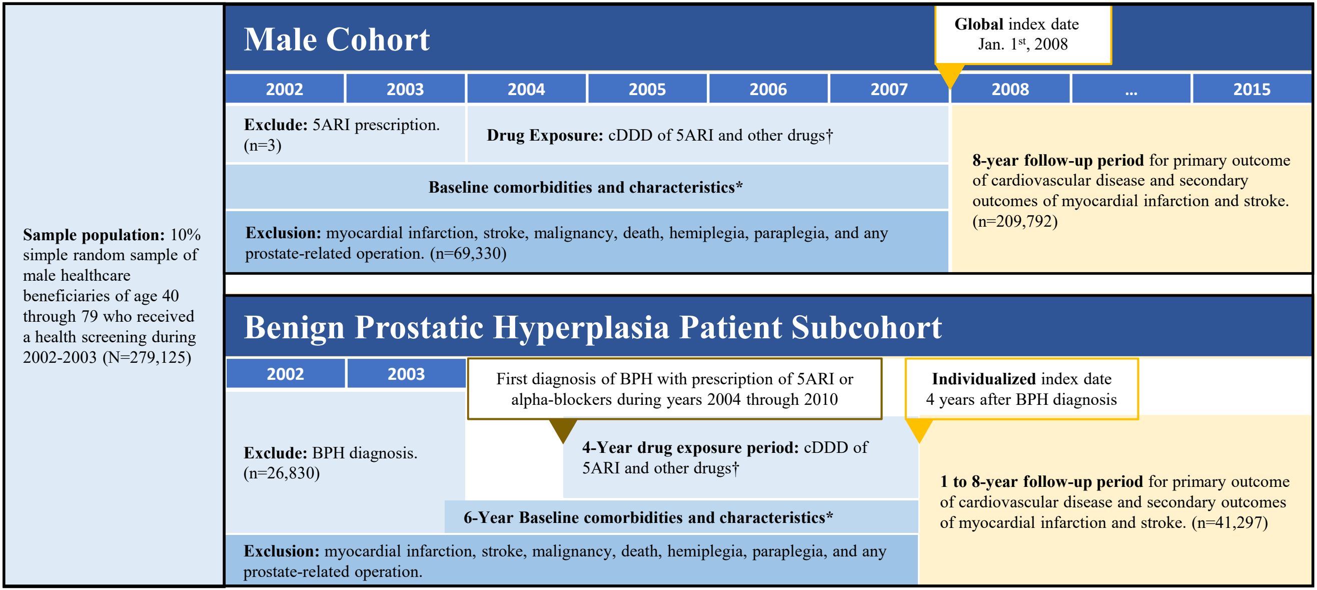

Abbreviations: 5ARI, 5-alpha reductase inhibitor; cDDD, cumulative daily defined dose; BPH, benign prostatic hyperplasia.
† Alpha-blocker, HMG-CoA reductase inhibitor, metformin, aspirin, and non-aspirin NSAID.
* Age, body mass index, smoking habit, alcohol consumption frequency, exercise frequency, quartiles of socioeconomic status, number of outpatient visits, angina, hypertension, diabetes, high cholesterol, acute urinary retention, benign prostatic hyperplasia, atrial fibrillation/flutter, Charleson Comorbidity Index (including congestive heart failure, peripheral vascular disease, dementia, pulmonary disease, connective tissue disorder, peptic ulcer, liver disease, renal disease, severe liver disease, and HIV).

Supplementary Table 2. Hazard ratios of 5ARI users vs. non-users after stratification by cardiovascular risk factors

		CVD (Stroke or MI)	Myocardial Infarction	Stroke
		aHR† (95% CI)	aHR† (95% CI)	aHR† (95% CI)
Main cohort	Age			
	<60 years	0.89 (0.64-1.23)	1.08 (0.60-1.97)	0.81 (0.55-1.20)
	≥60 years	1.13 (0.95-1.33)	1.14 (0.78-1.66)	1.12 (0.93-1.35)
	Hypertension			
	No	1.08 (0.89-1.31)	1.22 (0.82-1.83)	1.04 (0.83-1.30)
	Yes	1.03 (0.82-1.29)	0.91 (0.53-1.55)	1.06 (0.83-1.36)
	Diabetes			
	No	1.02 (0.86-1.21)	1.17 (0.82-1.69)	0.99 (0.81-1.20)
	Yes	1.19 (0.89-1.61)	1.01 (0.50-2.04)	1.24 (0.89-1.72)
BPH Patient Subcohort	Age			
	<60 years	1.03 (0.87-1.21)	0.93 (0.68-1.28)	1.06 (0.88-1.28)
	≥60 years	0.94 (0.86-1.03)	0.85 (0.68-1.05)	0.96 (0.87-1.05)
	Hypertension			
	No	0.96 (0.87-1.05)	0.94 (0.76-1.15)	0.96 (0.87-1.07)
	Yes	0.92 (0.81-1.06)	**0.67 (0.48-0.95)**	0.98 (0.84-1.13)
	Diabetes			
	No	0.94 (0.86-1.02)	0.87 (0.72-1.06)	0.95 (0.87-1.05)
	Yes	0.99 (0.83-1.18)	0.79 (0.53-1.18)	1.04 (0.85-1.27)

Abbreviations: 5ARI, 5-alpha reductase inhibitor; CVD, cardiovascular disease; MI, myocardial infarction; aHR, adjusted hazard ratio; ref., referent.

† Using a multivariate cox proportional hazard (Wald χ^2 test *P*-value<0.001) adjusted for age, hypertension, diabetes, high cholesterol, body mass index, smoking habit, alcohol consumption frequency, exercise frequency, socioeconomic status in quartiles, benign prostatic hyperplasia, acute urinary retention, atrial fibrillation or flutter, angina, Charlson Comorbidity Index, outpatient visits, alpha-blocker use, aspirin use, NSAID use, HMG-CoA reductase inhibitor use, and year of first 5ARI prescription or year of BPH diagnosis (for BPH patient subcohort).

Supplementary Table 3. Hazard ratios of tertiles of 5ARI users vs. non-users

	Exposure to 5ARI			
	Non-user	Q1 of user	Q2 of user	Q3 of user
Main cohort				
5ARI exposure, cDDD	0	1-30	31-130	≥131
CVD (Stroke or MI)				
Cases	10,101	276	227	241
aHR† (95% CI)	1(ref.)	1.12 (0.95-1.33)	1.05 (0.87-1.46)	0.86 (0.71-1.07)
Myocardial Infarction				
Cases	2,387	62	38	48
aHR† (95% CI)	1(ref.)	1.29 (0.90-1.86)	0.85 (0.62-1.46)	0.91 (0.56-1.48)
Stroke				
Cases	7,682	215	185	187
aHR† (95% CI)	1(ref.)	1.08 (0.90-1.31)	1.07 (0.87-1.31)	0.85 (0.67-1.08)
BPH patient subcohort				
5ARI exposure, cDDD	0	1-42	43-216	≥217
CVD (Stroke or MI)				
Cases	1,348	556	509	471
aHR† (95% CI)	1(ref.)	1.02 (0.93-1.13)	0.97 (0.88-1.07)	**0.81 (0.70-0.92)**
Myocardial Infarction				
Cases	285	110	85	87
aHR† (95% CI)	1(ref.)	1.01 (0.81-1.26)	0.83 (0.66-1.04)	**0.69 (0.50-0.95)**
Stroke				
Cases	1,067	446	424	386
aHR† (95% CI)	1(ref.)	1.02 (0.91-1.15)	1.00 (0.90-1.12)	**0.84 (0.72-0.98)**

Abbreviations: 5ARI, 5-alpha reductase inhibitor; cDDD, cumulative daily defined dose; CVD, cardiovascular disease; aHR, adjusted hazard ratio; ref., referent; Q, quantile(tertile).

† Using a multivariate cox proportional hazard (Wald χ^2 test P-value<0.001) adjusted for age, hypertension, diabetes, high cholesterol, body mass index, smoking habit, alcohol consumption frequency, exercise frequency, socioeconomic status in quartiles, acute urinary retention, atrial fibrillation or flutter, angina, Charlson Comorbidity Index, outpatient visits, alpha-blocker use, aspirin use, NSAID use, and HMG-CoA reductase inhibitor use. Main cohort was additionally adjusted for benign prostatic hyperplasia and year of first 5ARI prescription; BPH patient subcohort, the year of BPH diagnosis.
